# Linear Regression Equations To Predict β-Lactam, Macrolide, Lincosamide, and Fluoroquinolone MICs from Molecular Antimicrobial Resistance Determinants in *Streptococcus pneumoniae*

**DOI:** 10.1128/AAC.01370-21

**Published:** 2022-01-18

**Authors:** Walter Demczuk, Irene Martin, Averil Griffith, Brigitte Lefebvre, Allison McGeer, Gregory J. Tyrrell, George G. Zhanel, Julianne V. Kus, Linda Hoang, Jessica Minion, Paul Van Caeseele, Rita Raafat Gad, David Haldane, George Zahariadis, Kristen Mead, Laura Steven, Lori Strudwick, Michael R. Mulvey

**Affiliations:** a National Microbiology Laboratory, Public Health Agency of Canada, Winnipeg, Manitoba, Canada; b Laboratoire de Santé Publique du Québec, Sainte-Anne-de-Bellevue, Quebec, Canada; c Toronto Invasive Bacterial Diseases Network (TIBDN), Department of Microbiology, Mount Sinai Hospital, Toronto, Ontario, Canada; d The Provincial Laboratory for Public Health (Microbiology), Edmonton, Alberta, Canada; e Department of Medical Microbiology and Infectious Diseases, Faculty of Medicine, University of Manitoba, Winnipeg, Manitoba, Canada; f Public Health Ontario, Toronto, Ontario, Canada; g Department of Laboratory Medicine and Pathobiology, University of Toronto, Toronto, Ontario, Canada; h British Columbia Centre for Disease Control, Vancouver, British Columbia, Canada; i Roy Romanow Provincial Laboratory, Regina, Saskatchewan, Canada; j Cadham Provincial Laboratory, Winnipeg, Manitoba, Canada; k Office of the Chief Medical Officer of Health, New Brunswick Department of Health, Fredericton, New Brunswick, Canada; l Queen Elizabeth II Health Science Centre, Halifax, Nova Scotia, Canada; m Newfoundland and Labrador Public Health Laboratory, St. John’s, Newfoundland, Canada; n Queen Elizabeth Hospital, Charlottetown, Prince Edward Island, Canada; o Stanton Territorial Hospital Laboratory, Yellowknife, Northwest Territories, Canada; p Yukon Communicable Disease Control, Whitehorse, Yukon, Canada

**Keywords:** MIC, *Pneumococcus*, *Streptococcus pneumoniae*, antibiotic resistance, antimicrobial resistance, minimum inhibitory concentration, molecular biology, molecular subtyping

## Abstract

Antimicrobial resistance in Streptococcus pneumoniae represents a threat to public health, and monitoring the dissemination of resistant strains is essential to guiding health policy. Multiple-variable linear regression modeling was used to determine the contributions of molecular antimicrobial resistance determinants to antimicrobial MICs for penicillin, ceftriaxone, erythromycin, clarithromycin, clindamycin, levofloxacin, and trimethoprim-sulfamethoxazole. Training data sets consisting of Canadian S. pneumoniae isolates obtained from 1995 to 2019 were used to generate multiple-variable linear regression equations for each antimicrobial. The regression equations were then applied to validation data sets of Canadian (*n* = 439) and U.S. (*n* = 607 and *n* = 747) isolates. The MICs for β-lactam antimicrobials were fully explained by amino acid substitutions in motif regions of the penicillin binding proteins PBP1a, PPB2b, and PBP2x. Accuracies of predicted MICs within 1 doubling dilution to phenotypically determined MICs were 97.4% for penicillin, 98.2% for ceftriaxone, 94.8% for erythromycin, 96.6% for clarithromycin, 98.2% for clindamycin, 100% for levofloxacin, and 98.8% for trimethoprim-sulfamethoxazole, with an overall sensitivity of 95.8% and specificity of 98.0%. Accuracies of predicted MICs to the phenotypically determined MICs were similar to those of phenotype-only MIC comparison studies. The ability to acquire detailed antimicrobial resistance information directly from molecular determinants will facilitate the transition from routine phenotypic testing to whole-genome sequencing analysis and can fill the surveillance gap in an era of increased reliance on nucleic acid assay diagnostics to better monitor the dynamics of S. pneumoniae.

## INTRODUCTION

Streptococcus pneumoniae is a common Gram-positive microorganism of the human nasopharynx that can cause severe invasive pneumococcal diseases (IPDs), such as bacteremia and meningitis. In this environment, exposure to other commensal bacteria, host immune responses, and sublethal antimicrobial levels increase selective pressures and favor the exchange of genetic material (1–4). The pneumococcal genome also contains numerous insertion sequences and other mobile genetic elements that facilitate the uptake of antimicrobial resistance determinants through horizontal recombination events (2, 5). Due to the ease with which S. pneumoniae can acquire novel antimicrobial resistance determinants, prompt detection of their emergence, dissemination, and dynamics through surveillance systems is essential to public health. The implementation of pediatric vaccination programs has been successful not only in lowering general incidence of IPD, but also by targeting serotypes associated with antimicrobial resistance, achieving a concurrent decrease in overall antimicrobial resistance (6, 7). The proliferation of antimicrobial-resistant isolates of nonvaccine serotypes, however, remains a major global health concern (8, 9).

The antimicrobial resistance mechanisms of S. pneumoniae have been extensively documented and with few exceptions can fully explain the observed antimicrobial phenotypes (10, 11). β-Lactam resistance has been attributed to genetic changes in the transpeptidase domains of penicillin binding proteins (encoded by *pbp1a*, *pbp2b*, and *pbp2x*), macrolide and lincosamide resistance to the presence of 23S rRNA methyltransferases (encoded by *ermB* and *ermTR*), the ABC-efflux pump (encoded by *mefAE*), or the number of 23S rRNA alleles with point mutations in the peptidyltransferase loop of domain V, fluoroquinolone resistance due to genetic changes in quinolone resistance-determining regions (QRDR) of *gyrA* and/or *parC*, tetracycline resistance due to the presence of *tetM* or *tetO*, chloramphenicol resistance due to the presence of *cat*, and trimethoprim-sulfamethoxazole resistance caused by genetic mutations in *folA* and *folP* (10, 12–24).

Monitoring the dissemination and dynamics of antimicrobial resistance of S. pneumoniae has traditionally relied upon *in vitro* phenotypic susceptibility testing of bacterial cultures; however, the development of whole-genome sequencing and novel molecular-based techniques to determine antimicrobial resistance can increase efficiency and decrease the labor associated with screening and monitoring strains for surveillance purposes. A EUCAST subcommittee reviewed the existing data on the use of whole-genome sequencing for the prediction of antimicrobial resistance for a number of bacterial species (25). The committee concluded that there was a lack of studies on the utility of whole-genome sequencing for the prediction of antimicrobial resistance in antimicrobials used to treat S. pneumoniae infections. Multiple-variable linear regression modeling has been successfully used to accurately predict MICs of a variety of agents for Neisseria gonorrhoeae (26–28). In this study, we employ a computational statistical approach not only to directly determine antimicrobial MICs from molecular determinants, but also to determine the relative contribution of each determinant to the overall MIC and present simple mathematical equations that can be applied to determine penicillin, ceftriaxone, erythromycin, clarithromycin, clindamycin, levofloxacin, and trimethoprim-sulfamethoxazole MIC values for S. pneumoniae.

## RESULTS

Regression analysis indicated that the predicted MIC (MIC_pred_) for penicillin depended upon any alteration of the PBP1a-STMK, PBP1a-TSQF, PBP2b-SSNT, PBP2b-QLQPT, and/or PBP2x-LKSG motifs and specific alterations of PBP2x-STMK→SAFK and PBP2x-KDA→EDT or KEA. No isolates were found in the training or validation data sets to have modifications of the PBP1a-SSN, PBP1a-KTG, PBP2b-SVVK, PBP2b-KTG, or PBP2x-SSN motifs (Table 1).

**TABLE 1:** Distribution of penicillin MICs and penicillin binding protein motif profiles of isolates in the multiple-variable linear regression training data set^*a*^

PBP1a motifs	PBP2b motifs	PBP2x motifs	No. of isolates with penicillin MIC (mg/L) of:								
			0.03	0.06	0.125	0.25	0.5	1	2	4	8
**SSMK**/WT/WT/**NTGY**	WT/**SSNA**/**AIDTK**/WT	**SAFK**/WT/**KEA**/**VKSG**								2	2

**SSMK**/WT/WT/**NTGY**	WT/**SSNA**/**AIDTK**/WT	SAMK/WT/**EDT**/**VKSG**					1	2	7	17	6
**SSMK**/WT/WT/**NTGY**	WT/**SSNA**/**AIDTK**/WT	SAMK/WT/EDA/**VKSG**								1	

**SAMK**/WT/WT/**NTGY**	WT/**SSNA**/**TVDTK**/WT	SAMK/WT/**KEA**/**VKSG**								1	
**SAMK**/WT/WT/**NTGY**	WT/**SSNA**/**AIDTK**/WT	SAMK/WT/**KEA**/**VKSG**							1	1	
**SAMK**/WT/WT/**NTGY**	WT/**SSNA**/WT/WT	SAMK/WT/**KEA**/**VKSG**					1	8	28	6	

**SSMK**/WT/WT/**NTGY**	WT/**SSNA**/**SVESK**/WT	SAMK/WT/**KEA**/**VKSG**							1		
**SSMK**/WT/WT/**NTGY**	WT/**SSNA**/WT/WT	SAMK/WT/**KEA**/**VKSG**			1		3	11	11	1	
**SSMK**/WT/WT/**NTGY**	WT/**SSNA**/WT/WT	SAMK/WT/**EDT**/**VKSG**							1		

**SAMK**/WT/WT/**NTGY**	WT/**SSNA**/WT/WT	WT/WT/WT/WT		1		1	4				
**SAMK**/WT/WT/**NTGY**	WT/**SSNA**/**SVESK**/WT	WT/WT/WT/WT					1				
**SAMK**/WT/WT/**NTGY**	WT/**SSNA**/**SVESK**/WT	WT/WT/EDA/**VKSG**				1					
**SAMK**/WT/WT/**NTGY**	WT/**SSNA**/WT/WT	SPMK/WT/WT/WT			1						

**SSMK**/WT/WT/**NTGY**	WT/**SSNA**/**AIDTK**/WT	SAMK/WT/**KEA**/**VKSG**					1				
**SSMK**/WT/WT/**NTGY**	WT/**SSNS**/WT/WT	SAMK/WT/**KEA**/**VKSG**					1				
**SSMK**/WT/WT/**NTGY**	WT/WT/WT/WT	SAMK/WT/**KEA**/**VKSG**				1					
**SSMK**/WT/WT/**NTGY**	WT/**SSNA**/WT/WT	WT/WT/WT/WT					1				

WT/WT/WT/**NTGY**	WT/**SSNA**/WT/WT	SAMK/WT/**KEA**/**VKSG**				2	4		1		
WT/WT/WT/**NTGY**	WT/**SSNA**/**SVETK**/WT	SPMK/WT/WT/WT					1				
WT/WT/WT/**NTGY**	WT/**SSNA**/WT/WT	WT/WT/WT/WT		2	4	16	4				
WT/WT/WT/**NTGY**	WT/**SSNA**/**SVESK**/WT	WT/WT/WT/WT				1					
WT/WT/WT/**NTGY**	WT/**SSNA**/**SVESK**/WT	WT/WT/EDA/**VKSG**				3					
WT/WT/WT/**NTGY**	WT/**YSSN**/**QQLQP**/GKT	WT/WT/WT/**VKSG**				1					
WT/WT/WT/**TSQY**	WT/**YSSN**/**QQLQP**/GKT	WT/WT/WT/WT				1					
WT/WT/WT/**TSQY**	WT/**SSNA**/**SVESK**/WT	WT/WT/WT/WT				1					
WT/WT/WT/**TSQY**	WT/**SSNA**/WT/WT	SAMK/WT/WT/WT				1					

WT/WT/WT/**TSQY**	WT/**SSNA**/**SVESK**/WT	SAMK/WT/WT/WT			1						
WT/WT/WT/**NTGY**	WT/WT/WT/WT	WT/WT/WT/WT			1						
WT/WT/WT/**NTGY**	WT/WT/WT/WT	WT/WT/**KEA**/WT			1						
WT/WT/WT/**NTGY**	WT/WT/WT/WT	SAMK/WT/WT/WT	1	19	1						

WT/WT/WT/WT	WT/**SSNA**/WT/WT	SAMK/WT/**KEA**/**VKSG**				2					
WT/WT/WT/WT	WT/**SSNA**/WT/WT	WT/WT/WT/**VKSG**				1					
WT/WT/WT/WT	WT/**SSNA**/WT/WT	WT/WT/**KEA**/WT		4	16	6					
WT/WT/WT/WT	WT/**SSNA**/**TVDTK**/WT	WT/WT/WT/WT			1	1					
WT/WT/WT/WT	WT/**SSNA**/**SVESK**/WT	WT/WT/WT/WT		2	9	3					
WT/WT/WT/WT	WT/**SSNA**/WT/WT	SAMK/WT/WT/WT	2	9	12						
WT/WT/WT/WT	WT/**SSNA**/**SVESK**/WT	SAMK/WT/WT/WT		1	1						
WT/WT/WT/WT	WT/**SSNA**/WT/WT	WT/WT/KAA/WT		6	3						
WT/WT/WT/WT	WT/**SSNA**/WT/WT	WT/WT/WT/WT		1	3						

WT/WT/WT/WT	WT/WT/WT/WT	WT/WT/**KEA**/**VKSG**	1	1							
WT/WT/WT/WT	WT/WT/WT/WT	WT/WT/KET/**VKSG**	3	1	1						
WT/WT/WT/WT	WT/WT/WT/WT	WT/WT/**KEA**/WT		1							
WT/WT/WT/WT	WT/WT/WT/WT	SAMK/WT/KAA/WT	4	1							
WT/WT/WT/WT	WT/WT/WT/WT	SAMK/WT/WT/WT	2	1							

WT/WT/WT/WT	WT/WT/WT/WT	WT/WT/WT/WT	279	191	8	2	1				

Total			292	241	64	44	23	21	50	29	8

a Unaltered “wild-type” (WT) penicillin binding protein (PBP) motifs correspond to S. pneumoniae R6 (NCBI accession no. AE007317.1: 332863 to 335022, 1494216 to 1496273, and 302261 to 304513; locus tags spr0329, spr1517, and spr0304, respectively). PBP1a represents STMK/SSN/KTG/TSFQ, with amino acid start positions at 370/446/557/574, PBP2b represents SVVK/SSNT/QLQPT/KTG, with start positions at 386/443/565/615, and PBP2x represents STMK/SSN/KDA/LKSG, with start positions at 337/395/505/546 (Fig. 3). Bold font indicates motifs that significantly contribute to the predicted penicillin MIC value through regression analysis.

Equation 1 shows a penicillin MIC regression model:

(1) $$\begin{array}{l} \text{penicillin MIC (mg/L)}=2^{\wedge }[\text{round }(-4.61+\\ \left(1.547\times \text{ PBP1a }\left(\text{motif }1\right)-\text{STMK}\to \text{any}\right)+\\ \left(0.949\times \text{ PBP1a }\left(\text{motif }4\right)-\text{TSQF}\to \text{any}\right)+\\ \left(1.202\times \text{ PBP2b }\left(\text{motif }2\right)-\text{SSNT}\to \text{any}\right)+\\ \left(0.356\times \text{ PBP2b }\left(\text{motif }3\right)-\text{QLQPT}\to \text{any}\right)+\\ \left(1.626\times \text{ PBP2x }\left(\text{motif }1\right)-\text{STMK}\to \text{SAFK}\right)+\\ \left(1.548\times \text{ PBP2x }\left(\text{motif }3\right)-\text{KDA}\to \text{EDT}\right)+\\ \left(0.680\times \text{ PBP2x }\left(\text{motif }3\right)-\text{KDA}\to \text{KEA}\right)+\\ \left(0.753\times \text{ PBP2x }\left(\text{motif }4\right)-\text{LKSG}\to \text{VKSG}\right))] \end{array}$$

where each molecular determinant has a value of 1 if present or 0 if absent.

The molecular determinants having the largest effect on penicillin MIC were modifications to the PBP2x-STMK→SAFK, PBP2x-KDA→EDT, and PBP1a-STMK→SAMK/SSMK amino acid motifs producing an adjusted *R*^2^ value of 0.893 (see Table S1 in the supplemental material). The PBP2x-KDA→EDT motif with two amino acid changes had a regression coefficient over two times that of the PBP2x-KDA→KEA motif, corresponding to a 2-fold increased contribution to the MIC_pred_ increment value. The accuracy of the resultant penicillin MIC_pred_ values calculated from the regression equation within 1 doubling dilution to the overall phenotypically determined MIC (MIC_pheno_) values of the Canadian and U.S. data sets was 97.4% (1,748/1,794), with a sensitivity and specificity of 92.8% and 96.5%, respectively (Table 2). There were four (4/1,354 [0.3%]) very major interpretative errors (VMEs; i.e., predicted susceptible but phenotypically resistant) seen in the U.S. data sets, with MIC_pred_ values of ≤0.03 mg/liter but MIC_pheno_ values ranging from 2 to 8 mg/liter. Alignments of *pbp1a*, *pbp2b*, and *pbp2x* genes from these isolates did not reveal any other major nucleotide differences from other susceptible strains to explain the discrepancy.

**TABLE 2:** Accuracy between MICs determined by logistic regression of molecular antimicrobial resistance determinants and phenotypically determined MICs

Antimicrobial	Data set^*a*^	No. or % of isolates matching MIC dilution of^*b*^:								SENS (%)^*c*^	SPEC (%)^*c*^	PPV (%)^*c*^	NPV (%)^*c*^	% of MIC interpretive errors^*d*^		
		No. >−2	No. −2	No. −1	No. 0	No. +1	No. +2	No. >2	% ±1					MI	ME	VME
Penicillin	Canada	0	1	21	405	11	1	0	99.5	100	99.7	97.5	100	2.3	0	0
	USA-1	4	4	38	451	91	12	7	95.6	87.8	97.2	94.6	93.3	8.1	0.3	0.5
	USA-2	2	3	75	458	198	9	2	97.9	94.2	87.7	96.1	82.6	8.2	0.1	0.1
	All	7	8	133	1,314	300	22	9	97.4	92.8	96.5	95.8	93.6	6.7	0.1	0.2

Ceftriaxone	Canada	0	2	5	422	9	1	0	99.3	100	98.8	68.8	100	1.1	0	0
	USA-1	2	4	20	535	43	2	1	98.5	98.8	95.3	76.0	99.8	7.2	0	0.2
	USA-2	1	6	18	586	122	11	3	97.2	96.5	96.5	91.0	98.7	10.0	0	0.1
	All	3	12	43	1,543	174	14	4	98.2	97.2	96.7	85.2	99.5	6.9	0	0.1

Erythromycin	Canada	NA^*e*^	NA	NA	NA	NA	NA	NA	NA	NA	NA	NA	NA	NA	NA	NA
	USA-1	18	8	13	488	2	0	5	94.2	91.2	97.5	81.3	98.9	0.7	2.2	0.9
	USA-2	14	15	70	601	41	3	3	95.3	99.0	99.4	99.5	98.8	0.3	0.3	0.3
	All	32	23	83	1,089	43	3	8	94.8	98.1	98.3	97.1	98.9	0.5	1.1	0.6

Clarithromycin	Canada	2	1	10	368	46	5	7	96.6	100	100	100	100	0.2	0	0
	USA-1	NA	NA	NA	NA	NA	NA	NA	NA	NA	NA	NA	NA	NA	NA	NA
	USA-2	NA	NA	NA	NA	NA	NA	NA	NA	NA	NA	NA	NA	NA	NA	NA

Clindamycin	Canada	0	1	1	437	0	0	0	99.8	100	99.8	95.7	100	0.2	0	0
	USA-1	NA	NA	NA	NA	NA	NA	NA	NA	NA	NA	NA	NA	NA	NA	NA
	USA-2	13	3	366	360	0	2	3	97.2	97.8	97.7	93.3	99.3	0.7	1.7	0.4
	All	13	4	367	797	0	2	3	98.2	98.1	98.6	93.6	99.6	0.5	1.1	0.3

Levofloxacin	Canada	0	0	38	389	11	0	0	100	100	100	100	100	0	0	0
	USA-1	NA	NA	NA	NA	NA	NA	NA	NA	NA	NA	NA	NA	NA	NA	NA
	USA-2	0	0	42	702	3	0		100	100	100	100	100	0	0	0
	All	0	0	79	1,091	14	0	0	100	100	100	100	100	0	0	0

Trimethoprim-sulfamethoxazole	Canada	0	0	191	215	32	1	0	99.8	100	97.2	82.5	100	0.5	0	0
	USA-1	NA	NA	NA	NA	NA	NA	NA	NA	NA	NA	NA	NA	NA	NA	NA
	USA-2	0	1	121	511	101	11	2	98.1	95.8	97.8	96.5	97.4	4.8	0	0.3
	All	0	1	312	726	133	10	2	98.8	96.5	97.5	94.0	98.6	4.0	0	0.2

Overall		57	49	1,027	6,928	710	56	33	97.8	95.8	98.0	94.2	98.6	3.4	0.3	0.2

a Canadian validation data set, *n* = 439; USA-1 data set, *n* = 607 (35); USA-2 data set, *n* = 747 (10).

b Shown is the number of isolates with MIC_pred_ and MIC_pheno_ values that differ by the number of doubling dilutions indicated.

c SENS, SPEC, PPV, and NPV represent sensitivity, specificity, positive predictive value, and negative predictive value, respectively.

d Shown are the percentages of isolates with minor (MI), major (ME), and very major (VME) interpretative errors for susceptibilities. Detailed totals of minor errors based on interpretative value combinations are available in Table S39 in the supplemental material.

e NA, no phenotypic MIC available.

Ceftriaxone MIC regression modeling resulted in fewer molecular determinants where there were no contributions to MIC_pred_ from PBP2b motif changes, with only the PBP1a-STMK, PBP2x-STMK, PBP2x-KDA, and PBP2x-LKSG motifs having an influence, with an adjusted *R*^2^ value of 0.72 (see Table S3 in the supplemental material). The PBP2x-STMK→SAFK motif had the greatest magnitude, with a regression coefficient of 2.7, twice that of modifications to next most influential motif, PBP1a-STMK, which had a regression coefficient of 1.3.

Equation 2 shows the ceftriaxone MIC regression model:

(2) $$\begin{array}{l} \text{ceftriaxone MIC}(\text{mg/L)}=2^{\wedge }[\text{round }(-2.709+\\ \left(1.25\times \text{ PBP1a }\left(\text{motif 1}\right)-\text{STMK}\to \text{any}\right)+\\ \left(2.72\times \text{ PBP2x }\left(\text{motif 1}\right)-\text{STMK}\to \text{SAFK}\right)+\\ \left(0.76\times \text{ PBP2x }\left(\text{motif 3}\right)-\text{KDA}\to \text{EDT}\right)+\\ \left(0.989\times \text{ PBP2x }\left(\text{motif 4}\right)-\text{LKSG}\to \text{VKSG}\right))] \end{array}$$

where each molecular determinant has a value of 1 if present or 0 if absent.

The MIC_pred_ for ceftriaxone had an overall accuracy of 98.2% to 1 doubling dilution of the MIC_pheno_ of the combined validation data sets. The specificity (measure of susceptibility) was 96.7%, and sensitivity (measure of resistance) was 97.2%. The relatively large number of minor (MI) errors for both penicillin (*n* = 121 [6.7%]) and ceftriaxone (*n* = 124 [6.9%]) were due to a large number of MIC_pred_ values within 1 doubling dilution of the intermediate resistance interpretative breakpoints and the very broad CLSI intermediate resistance interpretative breakpoint range for penicillin, covering 3 doubling dilutions from 0.125 to 1 mg/liter.

Equation 3 shows the erythromycin MIC regression model:

(3) $$\begin{array}{l} \text{erythromycin MIC (mg/L)}=2^{\wedge }[\text{round }(-2.934+\\ \left(2.877\times 23\text{S rRNA-A2059G}\right)+\\ \left(1.080\times 23\text{S rRNA-C2611T}\right)+\\ \left(9.482\times \mathrm{ermB}\right)+\\ \left(5.540\times \mathrm{mefAE}\right)+\\ \left(1.357\times \mathrm{mefAE}\text{ promoter}\right)+\\ \left(0.707\times \mathrm{mefAE}\text{ intergenic}\right))] \end{array}$$

where *ermB* and *mefAE* molecular determinants have a value of 1 or 0 if present or absent, respectively, “23S rRNA-A2059G” and “23S rRNA-C2611T” are the number of alleles with the point mutation present, and “*mefAE* promoter” and “*mefAE* intergenic” have a value of 1 or 0, corresponding to the presence or absence of the −364T substitution or the 99-bp deletion in the intergenic region between *mefE* and *mel*, respectively.

Seven isolates within the validation data sets possessed a predicted dysfunctional *ermB* gene and were considered *ermB* negative for regression analysis calculations. Specifically, an ErmB-G41E amino acid substitution was found in five isolates, ErmB-G41K was detected in one isolate, and an *ermB* adenosine nucleotide deletion at position 629 creating a pseudogene was observed in two isolates (see supplemental validation Data Set S12 in the supplemental material).

The MIC_pred_ for erythromycin was greatly influenced by the presence of *ermB* (coefficient = 9.5), *mefAE* (coefficient = 5.5), and the A2059G point mutation of 23S rRNA (coefficient = 2.9 for each mutated allele), with lesser contributions from the C2611T 23S rRNA mutation, *mefAE*-346T mutation, and 99-bp intergenic deletion, to achieve an adjusted *R*^2^ value of 0.96 (see Table S4 in the supplemental material). Although the coefficient value for the 23S rRNA-A2059G mutation is relatively low compared to those of the *ermB* or *mefAE* determinants, when all four alleles carry the mutation, the magnitude of the coefficient increases considerably to 12. The erythromycin MIC_pred_ had an overall accuracy of 94.8% (1,215/1,281) within 1 doubling dilution of the erythromycin MIC_pheno_ of the validation data sets and 98% sensitivity and specificity.

Equation 4 shows the clarithromycin MIC regression model:

(4) $$\begin{array}{l} \text{clarithromycin MIC (mg/L)}=2^{\wedge }[\text{round }(-4.984+\\ \left(1.819\times 23\text{S rRNA-A2059G}\right)+\\ \left(1.246\times 23\text{S rRNA-C2611T}\right)+\\ \left(10.820\times \mathrm{ermB}\right)+\\ \left(5.577\times \mathrm{mefAE}\right)+\\ \left(0.833\times \mathrm{mefAE}\text{ promoter}\right)+\\ \left(0.950\times \mathrm{mefAE}\text{ intergenic}\right))] \end{array}$$

where *ermB* and *mefAE* molecular determinants have a value of 1 or 0 if present or absent, respectively, “23S rRNA-A2059G” and “23S rRNA-C2611T” are the number of alleles with the point mutation present, and “*mefAE* promoter” and “*mefAE* intergenic” have a value of 1 or 0, corresponding to the presence or absence of the −364T substitution or the 99-bp deletion in the intergenic region between *mefE* and *mel*, respectively.

The regression equation for the clarithromycin MIC_pred_ had determinant coefficients similar to those for erythromycin, with *ermB*, *mefAE*, and the A2059G 23S rRNA point mutations contributing the most to the overall MIC, with values of 10.8, 5.6, and 1.8, respectively, resulting in an adjusted *R*^2^ value of 0.98 (see Table S5 in the supplemental material). Overall MIC accuracy within 1 doubling dilution was 96.6% (424/439), with 100% sensitivity and specificity.

Equation 5 shows the clindamycin MIC regression model:

(5) $$\begin{array}{l} \text{clindamycin MIC (mg/L)}=2^{\wedge }[\text{round }(-\text{2}.\text{815 }+\\ \left(0.456\times 23\text{S rRNA-A2059G}\right)+\\ \left(9.048\times \mathrm{ermB}\right))] \end{array}$$

where the *ermB* molecular determinant has a value of 1 if present or 0 if absent, and the value of “23S rRNA-A2059G” is the number of alleles with the point mutation present.

The regression model for clindamycin resistance included primarily the presence of *ermB* and a minor contribution from the 23S rRNA-A2059G mutation and had an adjusted *R*^2^ value of 0.97 (see Table S6 in the supplemental material). The C2611T 23S rRNA mutation did not contribute to the model, as only one isolate was present in the training data carrying the mutation in all four alleles, yet only having a MIC_pheno_ value of ≤0.125 mg/liter. This determinant was also rare in the validation data, present in two isolates of the USA-1 data set, for which the MIC_pheno_ values were not available. This minimal complement of resistance determinants for clindamycin reflected the distribution of MIC_pheno_ values observed in the training (see Table S12 in the supplemental material) and validation data sets (see Tables S26 and S27 in the supplemental material), where MICs are polarized with extremely low or very high values. In the USA-2 data set, which had a maximum clindamycin MIC_pheno_ value of 2 mg/liter, 0.74% of the isolates had MIC_pheno_ values between 0.25 and 2 mg/liter, and of the 439 isolates in the Canadian validation data set, where testing included dilutions up to ≥64 mg/liter, there were no MIC_pheno_ values in the range from 0.5 to 16 mg/liter observed. There was a 98.2% (1,164/1,186) overall accuracy between MIC_pred_ and MIC_pheno_ values, with over 98% specificity and sensitivity.

Equation 6 shows the levofloxacin MIC regression model:

$$\begin{array}{l} \text{levofloxacin MIC (mg/L)}=2^{\wedge }[\text{round }(-0.218+\\ \left(2.028\times \text{ GyrA-S81F}\right)+\\ \left(1.564\times \text{ GyrA-S81Y}\right)+\\ \left(3.564\times \text{ GyrA-S81L}\right)+\\ \left(1.654\times \text{ ParC-S79 any}\right)+\\ \left(0.834\times \text{ ParC-D83 any}\right))] \end{array}$$

where each molecular determinant has a value of 1 if present or 0 if absent.

The MIC_pred_ for levofloxacin was predominantly dependent upon GyrA amino acid mutations S81L (*n* = 2) and S81F (*n* = 16), having regression coefficients of 3.6 and 2.0, respectively. Other determinants contributing to a lesser extent to the overall MIC_pred_ values included the GyrA-S81Y mutations (*n* = 1) and any mutations at ParC-S79 or ParC-D83. The adjusted *R*^2^ value of 0.460 (see Table S7 in the supplemental material) was very low for the model, likely due to very small number of isolates with resistance determinants and corresponding MIC_pheno_ values of ≥8 mg/liter (*n* = 11) compared to the very large number of isolates with no determinants and MIC_pheno_ values of ≤2 mg/liter (*n* = 942) within the training data. Despite the low adjusted *R*^2^ value and only two phenotypically levofloxacin-resistant isolates in the validation data, the accuracy of MIC_pred_ values compared to MIC_pheno_ values within 1 doubling dilution approached 100% (1,185/1,186), with 100% sensitivity and specificity. One isolate was missing the open reading frame corresponding to the *gyrA* gene in the genome assembly.

Equation 7 shows the trimethoprim-sulfamethoxaxole MIC regression model:

(7) $$\begin{array}{l} \text{trimethoprim-sulfamethoxazole (MIC mg/L)}=2^{\wedge }[\text{round }(-2.250+\\ \left(1.600\times \text{ FolA-I100L}\right)+\\ \left(2.526\times \text{FolP disruption}\right))] \end{array}$$

where each molecular determinant has a value of 1 if present or 0 if absent.

Regression modeling for trimethoprim-sulfamethoxazole resistance indicated that disruption of FolP had a regression coefficient of 2.5, suggesting a larger influence on overall MIC_pred_ than the I100L FolA determinant, which had a coefficient of 1.6. The adjusted *R*^2^ value was 0.798 (see Table S8 in the supplemental material) for the model, and there was 98.8% overall accuracy of MIC_pred_ and MIC_pheno_, with a specificity of 97.5% and sensitivity of 96.5%.

The absence or presence of a single molecular determinant for chloramphenicol, doxycycline, and tetracycline resistance was used to assign a MIC_pred_ value as less than or greater than the susceptible or resistant interpretation breakpoint, rather than using multiple-variable linear regression analysis. The presence of the *cat* gene was associated with a MIC_pred_ value of ≥8 mg/liter (see Table S15 in the supplemental material), which resulted in 99.8% accuracy with corresponding MIC_pheno_ values, with sensitivity, specificity, positive predictive value (PPV), and negative predicted value (NPV) of 53.3%, 100%, 100%, and 98.5%, respectively. The low sensitivity can be attributed to the very abrupt resistance breakpoint between susceptible and resistant at ≤4 mg/liter and ≥8 mg/liter resulting in a relatively large number of possible phenotyping errors that were phenotypically resistant strains (*n* = 21) without the *cat* gene among a relatively low overall number of phenotypically resistant isolates (*n* = 45). Similarly, tetracycline and doxycycline MICs were determined solely by the presence of *tetM* (*tetO* was not detected in this study), giving a MIC_pred_ value for tetracycline of ≥8 mg/liter and a value for doxycycline of ≥4 mg/liter (see Tables S16 and S17 in the supplemental material). The accuracy, sensitivity, specificity, positive predictive value, and negative predictive value for tetracycline MIC_pred_ were 97.6%, 96.2%, 98.1%, 94.1% and 98.8%, those for doxycycline were 98.4%, 95.1%, 99.5%, 95.1% and 99.5%, respectively.

## DISCUSSION

Multiple-variable linear regression analysis is a relatively simple, yet powerful tool to determine the dynamics of a specific variables among a complex series of other factors. Determinants of interest affecting the MIC were identified for each antimicrobial by summarizing the phenotypic MIC values and molecular determinant profiles (Table 1; see Tables S9 to S17 in the supplemental material), and the regression model was optimized by removing, combining, and adding back individual factors while examining the effect on the regression model metrics. This analytical strategy has been successfully used to validate predicted MICs from whole-genome sequence data of N. gonorrhoeae (26–28). In this study, multiple-variable linear regression modeling of molecular antimicrobial resistance determinants accurately predicted the MIC values for the β-lactam, macrolide, lincosamide, fluoroquinolone, and folate pathway inhibitor antimicrobials investigated. There was 98% (range, 94.2 to 100%) overall accuracy between the predicted and phenotypically derived MIC values, with 96% (range, 87.8 to 100%) sensitivity and 98% (range, 87.7 to 100%) specificity.

Resistance to β-lactam antimicrobials in S. pneumoniae is associated with changes to the transpeptidase domains of penicillin binding proteins PBP1a, PBP2b, and PBP2x, with particular focus on three amino acid motifs in each protein (24). It has been suggested that changes in PBP2b and PBP2x provide low-level resistance, while high level resistance is achieved with additional changes to PBP1a (29). Although increased resistance to β-lactams caused by altered PBP amino acid motifs has been extensively reported, multiple-variable linear regression analysis identified another possible amino acid motif in each protein that may contribute to overall MIC levels and was able to predict the relative contribution of each mutation to the overall MIC. Multiple-variable linear regression modeling for predicting penicillin MICs identified any changes to two motifs of PBP1a and PBP2b and specific changes to three motifs of PBP2x as significantly contributing to the MIC. The ceftriaxone MIC regression model was simpler, lacking the PBP2b motifs as contributing factors. Penicillin and ceftriaxone models included any changes to the PBP1a-STMK motif and PBP2x-STMK→SAFK, PBP2x-KDA→EDT, and LKSG→VKSG specific motif changes. The regression coefficients for these shared determinants were similar for both penicillin and ceftriaxone MICs, except for PBP2x-STMK→SAFK, which had a 2-fold greater effect on ceftriaxone MICs, reflecting the importance of this mutation to overall resistance reported in other studies (10, 24). The regression models for predicting β-lactam MICs had 98% accuracy to those derived phenotypically, with 0.1% major interpretative errors and 0.2% very major errors. The relatively large number of minor interpretative errors in both penicillin and ceftriaxone MICs could be due to a large number of MIC_pred_ values within 1 doubling dilution of the intermediate CLSI resistance interpretative breakpoints, which are very broad for penicillin, covering 3 doubling dilutions from 0.125 to 1 mg/liter. These findings are similar to those from a previous study, which used PBP allelic profiles as a PBP type library to associate phenotypic MICs with specific alleles, giving a similar 98% accuracy within 1 doubling dilution of the phenotypic MIC, with major and very major interpretative errors slightly larger at 3% and 2%, respectively (18).

**FIG 1:**
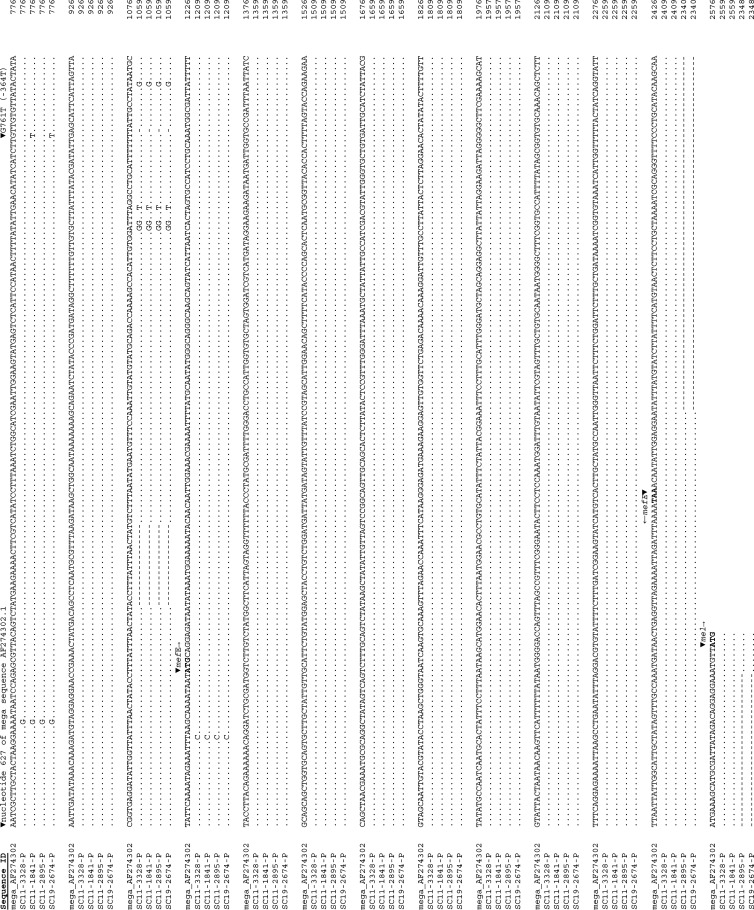
Alignment of representative DNA sequences of the macrolide efflux genetic assembly (mega) from *mefE* to *mel* of Streptococcus pneumoniae strains. The starting sequence position corresponds to position 627 of the mega sequence (GenBank accession no. AF274320). The start codons of *mefE* and *mel* are indicated. Sequence ID SC11-3328-P has a wild-type genotype and clarithromycin MIC of 2 mg/liter, SC11-1841-P has the −364T mutation and clarithromycin MIC of 4 mg/liter, SC11-2895-P has the 99-bp deletion in the intergenic region between *mefE* and *mel* and a clarithromycin MIC of 4 mg/liter, and SC19-2674-P has both the −364T substitution and the 99-bp deletion in the intergenic region between *mefE* and *mel* and a clarithromycin MIC of 8 mg/liter.

The greatest contributor to the macrolide and lincosamide MICs was the presence of *ermB*, which had similar regression coefficients for erythromycin, clarithromycin, and clindamycin, corresponding to 9 to 11 doubling MIC increments. The *mefAE* coefficients for erythromycin and clarithromycin were also similar, with increment values of about 5 for each antimicrobial, contributing about half as much as *ermB* to the MIC_pred_ values. The 23S rRNA-A2059G point mutation had regression coefficient values of 3 for each mutated allele for erythromycin and 2 for clarithromycin, but contributed much less to the clindamycin MIC, with a coefficient of only 0.5. The C2611T 23S rRNA resistance determinant contributed less than the A2059G determinant, with a value of about 1 for clarithromycin and erythromycin MICs; however, the C2611T mutation was not identified as a significantly contributing factor to increased clindamycin MICs. The G761T mutation, 364 nucleotides upstream (−364T) from the *mefE* start codon (Fig. 1), had a 2-fold greater influence upon the erythromycin predicted MIC than that for clarithromycin and had a similar influence to the 99-bp intergenic deletion between *mefE* and *mel*. Although the −364T mutation may be a considerable distance from the *mefE* start codon in the macrolide efflux genetic assembly to be located in the promoter region for *mefE*, there are a number of ATG start codons upstream before *mefE*, which may suggest that a small regulatory protein is located in this region. Accuracy with phenotypic MIC was best with clindamycin, with 98% accuracy, and both erythromycin and clarithromycin had accuracies of 95% and 97%, respectively, with sensitivities and specificities over 98% for all three antimicrobials. The USA-1 validation data set had an accuracy of 94% and a relatively low sensitivity of 91%, primarily due to 11 isolates with erythromycin MIC_pheno_ values of ≤0.5 mg/liter, despite having *mefAE* or an intact *ermB* as the sole resistance determinant, which should result in a MIC_pred_ value of ≥8 mg/liter, suggesting possible phenotyping errors. Conversely, there were 5 isolates in this data set that lacked any known molecular determinants but were phenotypically erythromycin resistant, having MICs of ≥1 mg/liter. Screening the discrepant genomes with additional molecular antimicrobial resistance determinant query tools ResFinder, ARG-ANNOT, and CARD (30–32) confirmed the genotypes. The observed discrepancies between molecular determinant profiles and expected resistance phenotypes may be due phenotypic reading errors, contamination, mislabeling, DNA sequencing errors, or possibly novel resistance mechanisms.

A single isolate in the training data set, and no isolates in the validation data sets, possessed the *ermTR* resistance determinant combined with *mefAE*. The single *ermTR*-positive isolate had MIC_pheno_ values for erythromycin and clarithromycin of ≥256 mg/liter and ≥16 mg/liter, respectively, similar to other isolates having an *ermB mefAE* genotype. Additional data are required to perform adequate regression analysis for the *ermTR* resistance determinant; however, speculatively its contribution to overall MIC_pred_ may be similar to that of *ermB*.

Fluoroquinolone resistance has been attributed to the GyrA-S81 and ParC-S79, -D83, and -N91 amino acid substitutions (10, 21). Regression modeling indicated that each of the three GyrA-S81F, -Y, and -L mutations had different contributions to the overall levofloxacin MIC, with the S81L mutation having about twice the effect of S81Y. Any mutation at ParC-D79 or -D83 significantly contributed the overall levofloxacin MIC_pred_; however, a D91 mutation was found in only a single isolate of the training data set, with a MIC_pheno_ value of 0.5 mg/liter (susceptible interpretation) and therefore did not contribute significantly during the modeling process. Despite have the lowest adjusted *R*^2^ value, the MIC_pred_ for levofloxacin had the best accuracy to MIC_pheno_ of all the antimicrobials analyzed, with accuracy to within 1 doubling dilution, all percentages of sensitivity and specificity of 100%, and no interpretive errors.

**FIG 2:**
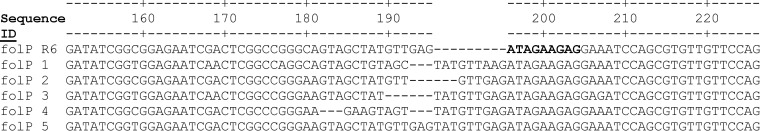
Alignment of representative S. pneumoniae *folP* DNA sequences showing insertion and deletion disruptions between nucleotide positions 151 and 226 in *folP* of S. pneumoniae R6 (GenBank accession no. NC_003098.1: 267995 to 268966, locus tag spr0266). Disrupted *folP* sequences associated with reduced susceptibility to trimethoprim-sulfamethoxazole are identified in those sequences where the amino acid motif at FolP positions 66 to 68 differ from the wild-type motif “IEE.” (Nucleotides corresponding to the motif are in bold.)

Molecular determinants for sulfamethoxazole-trimethoprim resistance include a simple and well-documented mechanism involving a FolA-I100L amino acid substitution and disruptions of *folP*, with a resistant phenotype observed when both determinants are present and intermediate when only one is present (10, 33, 34). MICs could be assigned simply as ≥4/76 mg/liter (resistant) when both determinants are present, 1/19 mg/liter or 2/38 mg/liter (intermediate) when only one is present, or ≤0.5/9.5 mg/liter (susceptible) when both determinants are wild type. A similar strategy can be used where the presence of *cat* in an isolate correlates with a chloramphenicol MIC_pred_ value of ≥8 mg/liter (resistant CLSI breakpoint), or *tetM* or *tetO* with a tetracycline MIC_pred_ value of ≥16 mg/liter. Regression analysis, however, offers insight into the relative magnitudes of each determinant, and in the future, the specific nucleotide insertions of *folP* may each be investigated to add further refinement to the predictive models to enhance the accuracy and precision of the MIC predictions. Comparison of the magnitudes of the regression coefficients indicates that the FolP disruptions (Fig. 2; see Table S8 in the supplemental material) have a greater influence on MIC than the FolA-I100L mutation.

There was low variability of MIC accuracy values between the validation data sets, suggesting the regression equations are robust and may be applied broadly across testing sites. A regression model developed for penicillin MICs from PBP types described by Metcalf et al. (10, 18, 35) as a simulated training data set (http://www.cdc.gov/streplab/mic-tables.html) generated a regression equation very similar to that attained using the Canadian training data (see Tables S1 and S2 in the supplemental material). The accuracy and precision of the predicted MICs may continually be improved over time, with larger, broader, and more current training data to address consistency of sampling, culturing methods, laboratory testing procedures, interpretation of phenotypic results, geographical variation, and the discovery of novel resistance determinants. Despite some discrepancies, the comparison of MIC_pred_ to MIC_pheno_ compares favorably to comparison studies of purely phenotypic results. A summary of an interlaboratory quality control program for pneumococcal serotyping and antimicrobial susceptibility testing involving reference laboratories participating in the International Circumpolar Surveillance program had an 97% overall accuracy of tests within 1 doubling dilution of the modal MIC, with erythromycin and clindamycin accuracies of 92% and 89%, respectively (36). Other quality assurance programs that have collated accuracy for phenotypic antimicrobial susceptibility testing included the Canadian National Gonococcal Antimicrobial Susceptibility Comparison Program (37), where the average MIC accuracy ranged from 85.6% to 98.9%, and a 2018 comparison of international antimicrobial proficiency panel results from various Caribbean and South American countries (38) reported an overall accuracy of >90% for some participants, while accuracy among other laboratories ranged from 60.0% to 82.4%.

Limitations of the study include that the accuracy and precision of the MIC prediction based on molecular determinants are largely limited by the training data used to generate the regression equations. The training data may include variability due to the subjective nature of phenotypic testing, where the same phenotypes may not always be observed on repeat testing, molecular resistance profile errors, and the possible presence of as-yet-unidentified resistance factors. Rare resistance determinants need to be present in the training data in sufficient quantities to generate meaningful statistics. While using a large training data set to develop the regression model can resolve some discrepancies, some rare resistance patterns, such as very high β-lactam resistance, are reliant on the availability of a relatively small number of isolates with this phenotype. Furthermore, there may also be some rare resistance determinants that were not present or were present in insufficient numbers to significantly influence the regression model, such as some of the reported PBP motifs, *ermTR*, or the 23S rRNA point mutations. These limitations can be reduced by increasing the size of the training data with isolates from varied regions of the world and regularly updating the regression models with newly discovered factors and updated coefficient values for currently identified factors. The MIC prediction models described here can be easily regenerated using the molecular markers discussed in this study with local training phenotypic data sets, which may be more applicable to individual laboratory testing environments. This approach also directly identifies the magnitude of antimicrobial resistance determinants specifically contributing to overall MIC without the need for continual curation of allelic databases that infer MIC values.

There is a need for surveillance systems that not only closely track the dissemination of known resistant strains, but also promptly detect novel antimicrobial resistant clones as they emerge to limit their expansion. Over the short term, molecular-based methods may primarily be used for surveillance purposes. As molecular-based genomic techniques become more comprehensive and broadly available to track lineages, antibiotic resistance, and virulence and fitness determinants, the MIC predicting strategy described here may provide a powerful tool to replace traditional phenotypic testing in clinical settings. Mathematical modeling to describe biological systems can fill a surveillance gap in an era of increased reliance on nucleic acid assay diagnostics to monitor the dynamics of S. pneumoniae, and the ability to acquire detailed antimicrobial resistance information directly from molecular information will enhance the monitoring of the dynamics of S. pneumoniae to effectively inform public health interventions to reduce the burden of disease.

## MATERIALS AND METHODS

### Training and validation data sets and antimicrobial susceptibility testing.

Training data sets (see supplemental training Data Sets S1 to S11 in the supplemental material) consisted of S. pneumoniae isolates collected in Canada from 1995 to 2019 for national surveillance purposes that had both phenotypic antimicrobial susceptibilities as well as molecular characterization data available. Isolates for the penicillin (*n* = 772), ceftriaxone (*n* = 772), erythromycin (*n* = 324), clarithromycin (*n* = 847), clindamycin (*n* = 1,356), levofloxacin (*n* = 1,446), trimethoprim-sulfamethoxazole (*n* = 1,207), tetracycline (*n* = 573), doxycycline (*n* = 938), and chloramphenicol (*n* = 824) MIC training data sets were selected to provide a broad range of MICs and well-characterized antimicrobial resistance determinants. An additional simulated training data set (*n* = 4,339) for penicillin MICs was generated from PBP types described by Metcalf et al. (10, 18, 35; http://www.cdc.gov/streplab/mic-tables.html).

Validation data (supplemental validation Data Set D12) included 439 Canadian S. pneumoniae isolates collected during 2019 for which both phenotypic antimicrobial susceptibility and molecular characterization results were available, as well as data previously reported for 534 isolates from Massachusetts, USA, during 2001 to 2007 (USA-1 data set) (39) and 747 isolates collected through a study of the Active Bacterial Core surveillance (ABCs), Centers for Disease Control and Prevention, Atlanta, GA, USA, during 2015 (USA-2 data set) (10).

Testing on the training and validation data sets of Canadian and U.S. isolates of their susceptibility to penicillin, ceftriaxone, erythromycin, clarithromycin, clindamycin, levofloxacin, trimethoprim-sulfamethoxazole, tetracycline, doxycycline, and chloramphenicol was done using the broth microdilution method according to Clinical and Laboratory Standards Institute (CLSI) guidelines (40, 41). Oral penicillin V and meningitis ceftriaxone resistance breakpoint interpretations were used.

### Molecular analysis.

**FIG 3:**
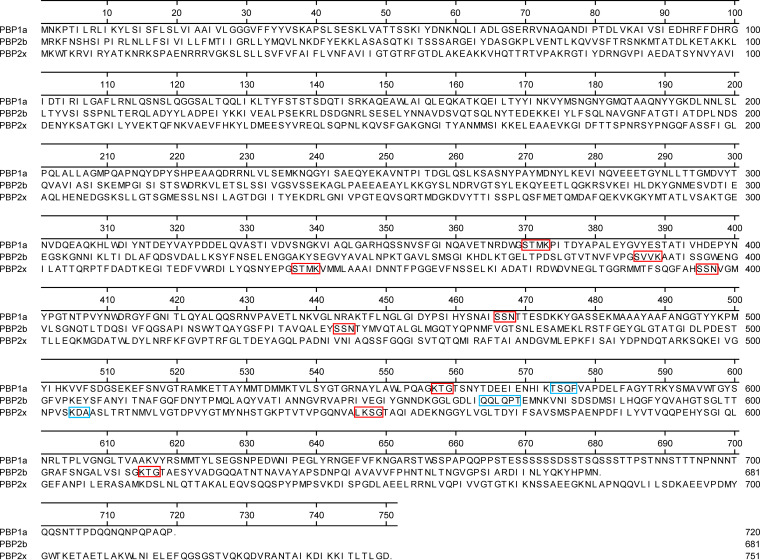
Amino acid alignment of penicillin binding proteins PBP1a, PBP2b, and PBP2x showing wild-type motifs associated with β-lactam resistance. PBP1a, PBP2b, and PBP2x are protein sequences from S. pneumoniae R6 (NCBI accession no. AE007317.1: 332863 to 335022, 1494216 to 1496273, and 302261 to 304513); locus tags spr0329, spr1517, and spr0304;, respectively. Amino acid sequences in red boxes are motifs previously described, and those in blue were identified as significantly contributing to increased β-lactam MICs through linear regression analysis.

Molecular antimicrobial resistance determinants were identified *in silico* from whole-genome sequencing data by querying reference (“wild-type”) gene nucleotide sequences against assembled contig files using BLAST (42), with the E value cutoff option set to 10e−100 and identifying relevant mutations or the presence or absence of the gene, as appropriate. Penicillin and ceftriaxone resistance determinants included changes to the “wild-type” amino acid SXXK, SXN, and KXG motifs in penicillin binding proteins PBP1a, PBP2b, and PBP2x from S. pneumoniae R6 (NCBI accession no. AE007317.1: 332863 to 335022, 1494216 to 1496273, and 302261 to 304513; locus tags spr0329, spr1517, and spr0304;, respectively) (14, 24). The wild-type PBP1a motifs STMK, SSN, and KTG had amino acid start positions 370, 446, and 557, respectively, the wild-type PBP2a motifs SVVK, SSNT, and KTG started at positions 386, 443 and 615, respectively, and the wild-type PBP2x motifs STMK, SSN, and LKSG started at positions 337, 395, and 546, respectively. An additional motif for each protein was identified through sequence alignment analysis of previously wild-type PBP motif profiles with relatively high MIC values. Novel motifs identified included TSQF, starting at position 574 of PBP1a, QLQPT, starting at position 565 of PBP2b, and KDA, starting at position 505 of PBP2x, bringing the total number of motifs analyzed per protein to four (Fig. 3).

Macrolide and lincosamide resistance determinants included the presence or absence of *ermB* (NCBI accession no. AB426620.1: 4320 to 5057), *ermTR* (CP002121.1: 856174 to 856905), *mefAE* (CP000921.1: 1802511 to 1803728); a G761T nucleotide mutation of the macrolide efflux genetic assembly (mega) sequence of GenBank accession no. AF274320.1 located in the *mefAE* promoter region 364 bp upstream [−364T] of the start codon (Fig. 1) (43), a 99-bp deletion in the intergenic region between *mefE* and *mel* (Fig. 1) (43), and 23S rRNA-A2059G and -C2611T point mutations (Escherichia coli numbering, corresponding to A2061G and C2613T in S. pneumoniae R6 GenBank accession no. AE007317.1, respectively). Alleles of *ermB* with G41E, G41K, or L63Q amino acid substitutions or an adenosine nucleotide insertion at position 628 conferred a susceptible macrolide phenotype to the strains and therefore were given an *ermB-*negative genotype. The number of 23S rRNA allele mutations was determined by a custom SNVPhyl workflow (44) using a 23S rRNA allele of S. pneumoniae R6 (GenBank accession no. AE007317.1, locus tag sprr02) as a mapping reference and interrogating the allele counts at nucleotide positions 2061 and 2613 from the resultant variant call files (.vcf). By convention, the locations of the 23S rRNA nucleotide mutations are based on the Escherichia coli coordinates of A2059G and C2611T (19, 20), which correspond to A2061G and C2613T, respectively, of S. pneumoniae R6.

Tetracycline resistance markers included the presence of *tetM* (AB426620.1: 14972 to 16891) or *tetO* (FM178797.1: 66 to 1985), and the chloramphenicol resistance determinant in the presence or absence of the *cat* gene (ICE6BST90: 56517 to 57167). The presence of the GyrA amino acid substitution S81 (AE007317.1: 1095463 to 1097931, locus tag spr1099) and ParC-S79, -D83, and -N91 substitutions (AE007317.1: 752250 to 754721, locus tag 0757) were analyzed as levofloxacin resistance determinants. Trimethoprim-sulfamethoxazole resistance determinants included the FolA-I100L (AE007317.1: 1412861 to 1413367) amino acid substitution and disruptions of *folP* (AE007317.1: 268022 to 268966) by nucleotide insertions or deletions in the region spanning nucleotide positions 169 to 196 corresponding to amino acid positions 57 to 66 (Fig. 2). Disrupted *folP* DNA sequences were detected by changes of the conserved “IEE” translated FolP amino acid motif at positions 66 to 68 indicating a shift in the protein reading frame.

### Multiple-variable linear regression analysis.

Multiple-variable linear regression analyses (45) were performed using Microsoft Excel 2010 (version 14.0.7151.5001; Microsoft Corp.) to determine the relationship of the molecular antimicrobial resistance determinants contained in an isolate to the phenotypically determined MIC value (MIC_pheno_) for each antimicrobial as previously described (27). The doubling MIC_pheno_ values were standardized to exact doubling dilutions (512, 256, 128, 64, 32, 16, 8, 4, 2, 1, 0.5, 0.25, 0.125, 0.0625, 0.03125, 0.015625, 0.0078125, 0.00390625, 0.001953125, and 0.000976563). The exact MICs were then converted to a linear increment scale using the formula phenotypic MIC increment = log_2_ (standardized MIC) and used as the dependent variable in the regression analysis. Molecular markers were used as independent variables and with presence or absence represented by a value of 1 and 0, respectively, except for the 23S rRNA-A2059G and -C2611T variables, which corresponded to the number of alleles with a respective mutation. A regression model for each antimicrobial was built from a preliminary analysis that included all independent variables followed by stepwise removal of variables with relatively high individual *P* values of >0.05 and those causing little change in the adjusted coefficient of determination (*R*^2^) value (Tables S1 to S8). To simplify the regression equations, variables with multiple possible mutations for a single resistance determinant having similar regression coefficients were collapsed into a single combined variable if the new regression coefficient was similar to the initial separately derived coefficients. For example, two of the possible PBP1a mutations for the STMK motif combined as “SAMK or SSMK” (“any”) had a similar coefficient value to each of the values calculated individually. Nonsignificantly contributing variables removed during the initial stepwise removal molecular determinants were then reintroduced to assess their contribution to the model.

An adjusted *R*^2^ value (95% confidence interval) of 0.0 to 0.1 was considered no correlation to very weak correlation, 0.2 to 0.4 was considered weak correlation, 0.5 to 0.7 was considered moderate correlation, 0.8 to 0.9 was considered strong correlation, and >0.9 was considered very strong correlation (27). Predicted MIC values (MIC_pred_) for each antimicrobial were calculated by first calculating the predicted MIC increment by summing the regression intercept and independent variable coefficients for each isolate, rounding fractional values up or down to the nearest whole integer and then converting this value back to a doubling MIC value using the formula predicted MIC value = 2^predicted MIC increment^. Individual *P* values of <0.05 for the independent variables at a confidence interval of 95% were considered significant.

Sensitivity (measure of resistance), specificity (measure of susceptibility), positive predictive value and negative predictive value for the MIC_pred_ were based on the accuracy to traditional MIC_pheno_ values: true positive (TP) having nonsusceptible (resistant or intermediate) predicted and phenotypic MICs, false negative (FN) having susceptible predicted MICs but nonsusceptible phenotypic MICs, true negative (TN) having susceptible predicted and phenotypic MICs, and false positive (FP) having a nonsusceptible predicted MICs and susceptible phenotypic MICs. Calculations were performed as follows: sensitivity (SENS) = TP/(FN + TP) × 100, specificity (SPEC) = TN/(FP + TN) × 100, positive predictive value = TP/(TP + FP), and negative predictive value = TN/(TN + FN) (46). Antimicrobial resistance interpretative errors were defined as minor error (MI) where the MIC_pred_ corresponded to intermediate resistance and the MIC_pheno_ corresponded to either susceptible or resistance interpretations and *vice versa*, major error (ME) where the MIC_pred_ corresponded to a resistant interpretation and the MIC_pheno_ was susceptible, and very major error (VME) where the MIC_pred_ was susceptible and MIC_pheno_ was resistant.
