# Supplementary material for: Linear Regression Equations To Predict β-Lactam, Macrolide, Lincosamide, and Fluoroquinolone MICs from Molecular Antimicrobial Resistance Determinants in Streptococcus pneumoniae
Source: Antimicrob Agents Chemother. 2022 Jan 18;66(1):e01370-21. doi: 10.1128/AAC.01370-21 (PMC8765234; doi:10.1128/AAC.01370-21)
Supplement: Supplemental file 1 — Tables S1 to S39. Download AAC.01370-21-s0001.pdf, PDF file, 0.3 MB [file aac.01370-21-s0001.pdf]

Supplemental Tables for

Linear regression equations to predict β-lactam, macrolide and fluoroquinolone minimum inhibitory concentrations from molecular antimicrobial resistance determinants in *Streptococcus pneumoniae*

Walter Demczuk, Irene Martin, Averil Griffith, Brigitte Lefebvre, Allison McGeer, Gregory J. Tyrrell, George G. Zhanel, Julianne V. Kus, Linda Hoang, Jessica Minion, Paul Van Caeseele, Rita Raafat Gad, David Haldane, George Zahariadis, Kristen Mead, Laura Steven, Lori Strudwick and Michael R. Mulvey

Regression Statistics Summary Output for Predicted Minimum Inhibitory Concentrations

Table S1. Penicillin.

SUMMARY OUTPUT								
Regression Statistics								
Multiple R	0.944854845							
R Square	0.892750679							
Adjusted R Square	0.891626177							
Standard Error	0.702410199							
Observations	772							
ANOVA								
	df	*SS*	*MS*	*F*	*Significance F*			
Regression	8	3133.587262	391.6984078	793.9080176	0			
Residual	763	376.4490073	0.493380088					
Total	771	3510.036269						
	Coefficients	*Standard Error*	*t Stat*	*P-value*	*Lower 95%*	*Upper 95%*	*Lower 95.0%*	*Upper 95.0%*
Intercept	-4.609850206	0.031127945	-148.0936266	0	-4.670956789	-4.548743624	-4.670956789	-4.548743624
pbp1a_1_ANY	1.546643583	0.155241106	9.962848263	4.60417E-22	1.241893187	1.851393978	1.241893187	1.851393978
pbp1a_4_ANY	0.949074251	0.102343152	9.273451428	1.81251E-19	0.748166663	1.14998184	0.748166663	1.14998184
pbp2b_2_ANY	1.202527621	0.092079775	13.05962814	2.52409E-35	1.021767842	1.383287399	1.021767842	1.383287399
pbp2b_3_ANY	0.356298924	0.137491208	2.591430605	0.009740468	0.086392962	0.626204887	0.086392962	0.626204887
pbp2x_1_SAFK	1.625871441	0.38126488	4.264414389	2.25578E-05	0.877418752	2.37432413	0.877418752	2.37432413
pbp2x_3_EDT	1.547626017	0.202003036	7.661399786	5.58574E-14	1.151078306	1.944173727	1.151078306	1.944173727
pbp2x_3_KEA	0.675981078	0.136776623	4.942226713	9.49902E-07	0.407477903	0.944484253	0.407477903	0.944484253
pbp2x_4_VKSG	0.753453309	0.157988047	4.769052622	2.2174E-06	0.443310454	1.063596164	0.443310454	1.063596164

Table S2. Penicillin using CDC PBP alleles as simulated training data.

SUMMARY OUTPUT								
Regression Statistics								
Multiple R	0.978745103							
R Square	0.957941976							
Adjusted R Square	0.9578448							
Standard Error	0.477128638							
Observations	4339							
ANOVA								
	df	*SS*	*MS*	*F*	*Significance F*			
Regression	10	22441.32849	2244.132849	9857.745343	0			
Residual	4328	985.2767173	0.227651737					
Total	4338	23426.60521						
	Coefficients	*Standard Error*	*t Stat*	*P-value*	*Lower 95%*	*Upper 95%*	*Lower 95.0%*	*Upper 95.0%*
Intercept	-4.950232674	0.008505566	-581.9992373	0	-4.96690794	-4.933557409	-4.96690794	-4.933557409
pbp1a_1_ANY	1.554309034	0.0500758	31.0391254	3.6915E-191	1.456134815	1.652483254	1.456134815	1.652483254
pbp1a_4_ANY	0.733542379	0.043218533	16.97286613	1.32789E-62	0.648811915	0.818272843	0.648811915	0.818272843
pbp2b_2_ANY	1.787981836	0.037560607	47.60258088	0	1.714343805	1.861619866	1.714343805	1.861619866
pbp2b_3_ANY	0.769270376	0.041675209	18.45870488	2.70181E-73	0.687565619	0.850975133	0.687565619	0.850975133
pbp2x_1_SAFK	1.194066015	0.08323012	14.34656121	1.2283E-45	1.030892344	1.357239686	1.030892344	1.357239686
pbp2x_1_SAMK	0.318802094	0.03696382	8.624706433	8.88869E-18	0.246334072	0.391270115	0.246334072	0.391270115
pbp2x_1_Other	0.978138266	0.145725869	6.712179999	2.16524E-11	0.692440913	1.263835619	0.692440913	1.263835619
pbp2x_3_EDT	1.328278639	0.069097168	19.2233442	4.29733E-79	1.192812795	1.463744483	1.192812795	1.463744483
pbp2x_3_KEA	0.955668367	0.050155959	19.05393473	8.60889E-78	0.857336994	1.053999739	0.857336994	1.053999739
pbp2x_4_VKSG	0.588672658	0.053970625	10.90727884	2.40694E-27	0.482862586	0.694482731	0.482862586	0.694482731

Table S3. Ceftriaxone.

SUMMARY OUTPUT								
Regression Statistics								
Multiple R	0.846390364							
R Square	0.716376649							
Adjusted R Square	0.714897518							
Standard Error	0.59877233							
Observations	772							
ANOVA								
	df	*SS*	*MS*	*F*	*Significance F*			
Regression	4	694.5735584	173.6433896	484.3226829	3.6901E-208			
Residual	767	274.9912084	0.358528303					
Total	771	969.5647668						
	Coefficients	*Standard Error*	*t Stat*	*P-value*	*Lower 95%*	*Upper 95%*	*Lower 95.0%*	*Upper 95.0%*
Intercept	-2.708629591	0.023839873	-113.6176189	0	-2.755428732	-2.661830449	-2.755428732	-2.661830449
pbp1a_1_ANY	1.252351165	0.11866248	10.55389339	2.08904E-24	1.019409394	1.485292936	1.019409394	1.485292936
pbp2x_1_SAFK	2.717006255	0.306330412	8.869528288	5.09935E-18	2.115660753	3.318351758	2.115660753	3.318351758
pbp2x_3_EDT	0.761123902	0.121454501	6.266741035	6.145E-10	0.522701223	0.999546582	0.522701223	0.999546582
pbp2x_4_VKSG	0.98927217	0.112363792	8.804189963	8.66835E-18	0.768695112	1.209849228	0.768695112	1.209849228

Table S4. Erythromycin.

SUMMARY OUTPUT								
Regression Statistics								
Multiple R	0.979394597							
R Square	0.959213777							
Adjusted R Square	0.958441798							
Standard Error	0.758950278							
Observations	324							
ANOVA								
	df	*SS*	*MS*	*F*	*Significance F*			
Regression	6	4294.255014	715.709169	1242.538723	6.8811E-217			
Residual	317	182.5937513	0.576005525					
Total	323	4476.848765						
	Coefficients	*Standard Error*	*t Stat*	*P-value*	*Lower 95%*	*Upper 95%*	*Lower 95.0%*	*Upper 95.0%*
Intercept	-2.93403471	0.051516945	-56.95280824	1.4867E-168	-3.035393046	-2.832676375	-3.035393046	-2.832676375
A2059G	2.876593457	0.078903938	36.45690593	2.0255E-115	2.721351879	3.031835035	2.721351879	3.031835035
C2611T	1.080210413	0.170408732	6.338938165	7.94726E-10	0.744935382	1.415485445	0.744935382	1.415485445
ermB	9.482421807	0.145721718	65.07212481	1.8571E-185	9.195717875	9.76912574	9.195717875	9.76912574
mefAE	5.540263267	0.145603887	38.05024296	3.233E-120	5.253791164	5.82673537	5.253791164	5.82673537
mefAEp	1.356954342	0.201939319	6.719614332	8.46333E-11	0.959643647	1.754265036	0.959643647	1.754265036
mefAEi	0.706650831	0.189512094	3.72879016	0.000227852	0.333790399	1.079511263	0.333790399	1.079511263

Where mefAEp represents the presence of the -364T mutation and mefAEi the 99 nucleotide deletion between *mefE* and *mel*.

Table S5. Clarithromycin.

SUMMARY OUTPUT								
Regression Statistics								
Multiple R	0.988792061							
R Square	0.977709739							
Adjusted R Square	0.977550523							
Standard Error	0.530601439							
Observations	847							
ANOVA								
	df	*SS*	*MS*	*F*	*Significance F*			
Regression	6	10373.15634	1728.859391	6140.769931	0			
Residual	840	236.4918251	0.281537887					
Total	846	10609.64817						
	Coefficients	*Standard Error*	*t Stat*	*P-value*	*Lower 95%*	*Upper 95%*	*Lower 95.0%*	*Upper 95.0%*
Intercept	-4.983620328	0.022417294	-222.3114124	0	-5.027620816	-4.939619839	-5.027620816	-4.939619839
A2059G	1.819193616	0.064617918	28.15308328	2.4225E-123	1.692362077	1.946025156	1.692362077	1.946025156
C2611T	1.245905082	0.132768695	9.384027474	5.77623E-20	0.985307733	1.50650243	0.985307733	1.50650243
ermB	10.81998396	0.074976035	144.3125665	0	10.67282159	10.96714633	10.67282159	10.96714633
mefAE	5.576783062	0.056344791	98.97601834	0	5.466189951	5.687376174	5.466189951	5.687376174
mefAEp	0.832666933	0.105732141	7.875248943	1.04928E-14	0.625136719	1.040197147	0.625136719	1.040197147
mefAEi	0.949820072	0.073489359	12.92459328	5.54592E-35	0.805575738	1.094064406	0.805575738	1.094064406

Where mefAEp represents the presence of the -364T mutation and mefAEi the 99 nucleotide deletion between *mefE* and *mel*.

Table S6. Clindamycin.

SUMMARY OUTPUT								
Regression Statistics								
Multiple R	0.982157747							
R Square	0.96463384							
Adjusted R Square	0.964581562							
Standard Error	0.459552127							
Observations	1356							
ANOVA								
	df	*SS*	*MS*	*F*	*Significance F*			
Regression	2	7793.668765	3896.834382	18451.95499	0			
Residual	1353	285.7375775	0.211188158					
Total	1355	8079.406342						
	Coefficients	*Standard Error*	*t Stat*	*P-value*	*Lower 95%*	*Upper 95%*	*Lower 95.0%*	*Upper 95.0%*
Intercept	-2.814504373	0.0130123	-216.2956872	0	-2.840030848	-2.788977899	-2.840030848	-2.788977899
A2059G	0.45590379	0.052490927	8.685382752	1.06892E-17	0.352931349	0.558876232	0.352931349	0.558876232
ermB	9.047514082	0.047113591	192.0361826	0	8.955090462	9.139937702	8.955090462	9.139937702

Table S7. Levofloxacin.

SUMMARY OUTPUT								
Regression Statistics								
Multiple R	0.679396034							
R Square	0.461578971							
Adjusted R Square	0.459709453							
Standard Error	0.458996807							
Observations	1446							
ANOVA								
	df	*SS*	*MS*	*F*	*Significance F*			
Regression	5	260.0793205	52.01586409	246.8973838	1.1716E-190			
Residual	1440	303.3764195	0.210678069					
Total	1445	563.45574						
	Coefficients	*Standard Error*	*t Stat*	*P-value*	*Lower 95%*	*Upper 95%*	*Lower 95.0%*	*Upper 95.0%*
Intercept	-0.217648329	0.012191394	-17.8526206	1.48695E-64	-0.241563122	-0.193733535	-0.241563122	-0.193733535
gyrA_S81F	2.028426081	0.150993315	13.43388001	7.52622E-39	1.732235667	2.324616494	1.732235667	2.324616494
gyrA_S81Y	1.563730177	0.480529622	3.254180608	0.00116359	0.621117141	2.506343213	0.621117141	2.506343213
gyrA_S81L	3.563730177	0.354358128	10.05686027	4.83675E-23	2.868616753	4.258843601	2.868616753	4.258843601
parC_S79	1.653918152	0.142386586	11.61568796	7.03318E-30	1.374610808	1.933225495	1.374610808	1.933225495
parC_D83	0.834456295	0.174679261	4.7770771	1.96018E-06	0.491803229	1.177109361	0.491803229	1.177109361

Table S8. Trimethoprim/Sulfamethoxazole.

SUMMARY OUTPUT

Regression Statistics	
Multiple R	0.893678694
R Square	0.798661608
Adjusted R Square	0.798327159
Standard Error	0.745331801
Observations	1207

ANOVA

	df	*SS*	*MS*	*F*	*Significance F*
Regression	2	2653.151216	1326.575608	2387.991106	0
Residual	1204	668.8454695	0.555519493		
Total	1206	3321.996686			

	Coefficients	*Standard Error*	*t Stat*	*P-value*	*Lower 95%*	*Upper 95%*	*Lower 95.0%*	*Upper 95.0%*
Intercept	-2.250333775	0.024755331	-90.9029968	0	-2.298902156	-2.201765393	-2.298902156	-2.201765393
folA	1.600207489	0.07775424	20.58032453	7.02014E-81	1.447658626	1.752756351	1.447658626	1.752756351
folP	2.525651954	0.062015606	40.72607082	1.1113E-228	2.403981288	2.647322619	2.403981288	2.647322619

Table S9. Ceftriaxone Phenotypic MICs and Distribution of Molecular Determinants in Training Dataset.

Molecular Determinant			Phenotypic MIC (mg/L)							
pbp1a motifs	pbp2b motifs	pbp2x motifs	0.125	0.25	0.5	1	2	4	8	Total
**SSMK**/WT/WT/NTGY	WT/SSNA/AIDTK/WT	**SAFK**/WT/KEA/**VKSG**						3	1	4
**SSMK**/WT/WT/NTGY	WT/SSNA/AIDTK/WT	SAMK/WT/**EDT**/**VKSG**	1		2	16	13	1		33
**SAMK**/WT/WT/NTGY	WT/SSNA/WT/WT	SAMK/WT/KEA/**VKSG**	1	3	2	31	6			43
**SAMK**/WT/WT/NTGY	WT/SSNA/TVDTK/WT	SAMK/WT/KEA/**VKSG**					1			1
**SAMK**/WT/WT/NTGY	WT/SSNA/AIDTK/WT	SAMK/WT/KEA/**VKSG**			1		1			2
**SSMK**/WT/WT/NTGY	WT/SSNA/WT/WT	SAMK/WT/KEA/**VKSG**	1	1	11	13	1			27
**SSMK**/WT/WT/NTGY	WT/SSNA/AIDTK/WT	SAMK/WT/EDA/**VKSG**				1				1
**SSMK**/WT/WT/NTGY	WT/SSNA/WT/WT	SAMK/WT/**EDT**/**VKSG**				1				1
**SSMK**/WT/WT/NTGY	WT/WT/WT/WT	SAMK/WT/KEA/**VKSG**				1				1
**SAMK**/WT/WT/NTGY	WT/SSNA/SVESK/WT	WT/WT/EDA/**VKSG**	1							1
**SAMK**/WT/WT/NTGY	WT/SSNA/SVESK/WT	WT/WT/WT/WT	1							1
**SAMK**/WT/WT/NTGY	WT/SSNA/WT/WT	SPMK/WT/WT/WT	1							1
**SAMK**/WT/WT/NTGY	WT/SSNA/WT/WT	WT/WT/WT/WT	4	2						6
**SSMK**/WT/WT/NTGY	WT/SSNS/WT/WT	SAMK/WT/KEA/**VKSG**			1					1
**SSMK**/WT/WT/NTGY	WT/SSNA/SVESK/WT	SSMK/WT/KEA/**VKSG**			1					1
**SSMK**/WT/WT/NTGY	WT/SSNA/AIDTK/WT	SAMK/WT/KEA/**VKSG**		1						1
**SSMK**/WT/WT/NTGY	WT/SSNA/WT/WT	WT/WT/WT/WT		1						1
WT/WT/WT/NTGY	WT/SSNA/WT/WT	SAMK/WT/KEA/**VKSG**		3	4					7
WT/WT/WT/NTGY	WT/SSNA/WT/WT	WT/WT/WT/WT	11	15						26
WT/WT/WT/NTGY	WT/SSNA/SVESK/WT	WT/WT/EDA/**VKSG**	2	1						3
WT/WT/WT/NTGY	WT/SSNA/SVESK/WT	WT/WT/WT/WT	1							1
WT/WT/WT/NTGY	WT/SSNA/SVETK/WT	SPMK/WT/WT/WT	1							1
WT/WT/WT/NTGY	WT/WT/WT/WT	SAMK/WT/WT/WT	20	1						21
WT/WT/WT/NTGY	WT/WT/WT/WT	WT/WT/WT/WT		1						1
WT/WT/WT/NTGY	WT/WT/WT/WT	WT/WT/KEA/WT	1							1
WT/WT/WT/NTGY	WT/YSSN/QQLQP/GKT	WT/WT/WT/**VKSG**	1							1
WT/WT/WT/TSQY	WT/SSNA/SVESK/WT	SAMK/WT/WT/WT	1							1
WT/WT/WT/TSQY	WT/SSNA/SVESK/WT	WT/WT/WT/WT	1							1
WT/WT/WT/TSQY	WT/SSNA/WT/WT	SAMK/WT/WT/WT	1							1
WT/WT/WT/TSQY	WT/YSSN/QQLQP/GKT	WT/WT/WT/WT	1							1
WT/WT/WT/WT	WT/SSNA/SVESK/WT	SAMK/WT/WT/WT	2							2
WT/WT/WT/WT	WT/SSNA/SVESK/WT	WT/WT/WT/WT	12	2						14
WT/WT/WT/WT	WT/SSNA/TVDTK/WT	WT/WT/WT/WT		2						2
WT/WT/WT/WT	WT/SSNA/WT/WT	SAMK/WT/KEA/**VKSG**	1		1					2
WT/WT/WT/WT	WT/SSNA/WT/WT	SAMK/WT/WT/WT	22	1						23
WT/WT/WT/WT	WT/SSNA/WT/WT	WT/WT/KAA/WT	5	4						9
WT/WT/WT/WT	WT/SSNA/WT/WT	WT/WT/KEA/WT	26							26
WT/WT/WT/WT	WT/SSNA/WT/WT	WT/WT/WT/**VKSG**		1						1
WT/WT/WT/WT	WT/SSNA/WT/WT	WT/WT/WT/WT	2	2						4
WT/WT/WT/WT	WT/WT/WT/WT	SAMK/WT/KAA/WT	4	1						5
WT/WT/WT/WT	WT/WT/WT/WT	SAMK/WT/WT/WT	2	1						3
WT/WT/WT/WT	WT/WT/WT/WT	WT/WT/KEA/**VKSG**	1	1						2
WT/WT/WT/WT	WT/WT/WT/WT	WT/WT/KEA/WT	1							1
WT/WT/WT/WT	WT/WT/WT/WT	WT/WT/KET/**VKSG**	5							5
WT/WT/WT/WT	WT/WT/WT/WT	WT/WT/WT/WT	325	150	5	1				481
Total			459	194	28	64	22	4	1	772

Note: Unaltered, "wild type" (WT) motif in *S. pneumoniae* R6 (GenBank accession number AE007317.1): PBP1a = STMK/SSN/KTG/TSFQ; PBP2b = SVVK/SSNT/QLQPT/KTG; PBP2x = STMK/SSN/KDA/LKSG. Bold font indicates motifs that significantly contribute to the predicted penicillin MIC value through regression analysis

Table S10. Erythromycin Phenotypic MICs and Distribution of Molecular Determinants in Training Dataset.

Molecular Determinant							Phenotypic MIC (mg/L)												
23S rRNA A2059G	23S rRNA C2611T	ermB	ermTR	mefAE	-364T	Del	0.125	0.25	0.5	1	2	4	8	16	32	64	128	256	Total
0	0	+	-	-	NA	NA									11	5	2	13	31
0	0	-	-	+	+	+							2	5	2	2			11
0	0	-	-	+	+	-							1	6	2	1			10
0	0	-	-	+	-	+						2	6	9	1				18
0	0	-	-	+	-	-				1	5	7	8	6					27
4	0	-	-	-	NA	NA												5	5
2	0	-	-	-	NA	NA					1			2				1	4
0	4	-	-	-	NA	NA					1								1
0	2	-	-	-	NA	NA				1									1
0	0	-	-	-	NA	NA	211	1	2	2									216
Total							211	1	2	4	7	9	17	28	16	8	2	19	324

Note: 23S rRNA 2059G and C2611T are the number of alleles with the mutation; *ermB*, *ermTR* and *mefAE* "-" or "+" corresponds to absence or presence of genes; -364T and Del represent the presence of the G761T (-364T from the start of *mefE*) substitution in the mega element (GenBank Accession AF274302.1), and the 99 base pair deletion in the intergenic region between *mefE* and *mel*, respectively.

Table S11. Clarithromycin Phenotypic MICs and Distribution of Molecular Determinants in Training Dataset.

Molecular Determinant							Phenotypic MIC (mg/L)												
23S rRNA A2059G	23S rRNA C2611T	ermB	ermTR	mefAE	-364T	Del	0.03125	0.0625	0.125	0.25	0.5	1	2	4	8	16	32	64	Total
0	0	+	-	-	NA	NA											9	46	55
0	0	-	-	+	+	+								1	2		1		4
0	0	-	-	+	+	-						5	11	9	2				27
0	0	-	-	+	-	+				1	3	6	25	55	2	1			93
0	0	-	-	+	-	-			1	3	8	25	50	14	1				102
4	0	-	-	-	NA	NA								2	2				4
0	4	-	-	-	NA	NA						1							1
2	0	-	-	-	NA	NA			1										1
0	0	-	-	-	NA	NA	553	5	1	1									560
Total							553	5	3	5	11	37	86	81	9	1	10	46	847

Note: 23S rRNA 2059G and C2611T are the number of alleles with the mutation; *ermB*, *ermTR* and *mefAE* "-" or "+" corresponds to absence or presence of genes; -364T and Del represent the presence of the G761T (-364T from the start of *mefE*) substitution in the mega element (GenBank Accession AF274302.1), and the 99 base pair deletion in the intergenic region between *mefE* and *mel*, respectively.

Table S12. Clindamycin Phenotypic MICs and Distribution of Molecular Determinants in Training Dataset.

Molecular Determinant					Phenotypic MIC (mg/L)								
23S rRNA A2059G	23S rRNA C2611T	ermB	ermTR	mefAE	0.125	0.25	0.5	1	4	32	64	128	Total
0	0	+	-	+						3	5	23	31
0	0	+	-	-					1	24	15	32	72
4	0	-	-	-			3	1					4
3	0	-	-	-		1							1
0	0	-	+	+		1							1
0	4	-	-	-	1								1
2	0	-	-	-	1								1
0	0	-	-	+	244	21	1						266
0	0	-	-	-	771	208							979
Total					1017	231	4	1	1	27	20	55	1356

Note: 23S rRNA 2059G and C2611T are the number of alleles with the mutation; *ermB*, *ermTR* and *mefAE* "-" or "+" corresponds to absence or presence of genes.

Table S13. Levofloxacin Phenotypic MICs and Distribution of Molecular Determinants in Training Dataset

Molecular Determinant				Phenotypic MIC (mg/L)								
GyrA S81	ParC S79	ParC D83	ParC N91	0.5	1	2	4	8	16	32	64	Total
S81L	S79F	WT	WT							2		2
S81F	S79F	WT	WT						3	1	1	8
S81F	S79Y	WT	WT						2			2
S81F	S79F	D83Y	WT						1			1
S81Y	S79Y	WT	WT					1				1
S81F	WT	WT	WT		3	1						5
WT	S79F	WT	WT		1	4						5
WT	S79Y	WT	WT		1	1						2
WT	WT	D83G	WT		1	1						2
WT	WT	D83N	WT		1	1						2
WT	WT	D83Y	WT			2						2
WT	WT	WT	N91S	1								1
WT	WT	WT	WT	320	1075	18						1413
Total				321	1082	28	0	0	6	3	1	1446

NOTE: "WT" denotes unaltered, wild type gene.

Table S14. Trimethoprim/Sulfamethoxazole Phenotypic MICs and Distribution of Molecular Determinants in Training Dataset.

Molecular Determinant		Phenotypic MIC (mg/L)						
FolA	FolP	0.125	0.25	0.5	1	2	4	Total
I100L	INS				1	10	136	147
WT	INS			19	82	44	5	150
I100L	WT		8	5	3	1	1	18
WT	WT	412	285	195				892
Total		412	293	219	86	55	142	1207

NOTE: "WT" denotes unaltered, wild type gene.

Table S15. Chloramphenicol Phenotypic MICs and Distribution of Molecular Determinants in Training Dataset.

Molecular Determinant	Phenotypic MIC (mg/L)						
cat	1	2	4	8	16	32	Total
-	151	362	292				805
+				4	9	6	19
Total	151	362	292	4	9	6	824

NOTE: "+" or "-" corresponds to the presence or absence of the gene, respectively.

Table S16. Tetracycline Phenotypic MICs and Distribution of Molecular Determinants in Training Dataset.

Molecular Determinant		Phenotypic MIC (mg/L)						
tetM	tetO	1	2	4	8	16	64	Total
-	-	380	128					508
+	-		3	2	3	56	1	65
Total		380	131	2	3	56	1	573

NOTE: "+" or "-" corresponds to the presence or absence of the gene, respectively.

Table S17. Doxycycline Phenotypic MICs and Distribution of Molecular Determinants in Training Dataset.

Molecular Determinant		Phenotypic MIC (mg/L)								
tetM	tetO	0.25	0.5	1	2	4	8	16	32	Total
-	-	778	6							784
+	-		3	16	44	42	21	27	1	154
Total		778	9	16	44	42	21	27	1	938

NOTE: "+" or "-" corresponds to the presence or absence of the gene, respectively.

Table S18. Penicillin predicted and phenotypic MIC accuracy on the Canadian validation dataset.

Predicted MIC (mg/L)	Phenotypic MIC (mg/L)								
	Susceptible		Intermediate				Resistant		
	≤0.03	0.06	0.12	0.25	0.5	1	2	4	Total
≤0.03	382	6							388
0.06	4	6							10
0.125	1		6	3	1				11
0.25			2	4	2				8
0.5				5	1				6
1									0
2						9	5		14
≥4							1	1	2
Total	387	12	8	12	4	9	6	1	439

Dark green highlight indicates matching MIC values; light green highlight indicates matching MIC values within +/- 1 dilution.

CLSI oral penicillin V resistance interpretative breakpoints used.

Table S19. Penicillin predicted and phenotypic MIC accuracy on the USA-1 validation dataset.

Predicted MIC (mg/L)	Phenotypic MIC (mg/L)									
	Susceptible		Intermediate				Resistant			
	≤0.03	0.06	0.12	0.25	0.5	1	2	4	8	Total
≤0.03	324	47	10	3		1	1		2	388
0.06	2	2	10							14
0.125	2	3	36	5						46
0.25	2	2	5	24	10					43
0.5				4	3					7
1					5	1				6
2		2				16	52	14	2	86
≥4							3	9	5	17
Total	330	56	61	36	18	18	56	23	9	607

Dark green highlight indicates matching MIC values; light green highlight indicates matching MIC values within +/- 1 dilution.

CLSI oral penicillin V resistance breakpoints used.

Table S20. Penicillin predicted and phenotypic MIC accuracy on the USA-2 validation dataset.

Predicted MIC (mg/L)	Phenotypic MIC (mg/L)									
	Susceptible		Intermediate				Resistant			
	≤0.03	0.06	0.12	0.25	0.5	1	2	4	8	Total
≤0.03	134	21	9					1		165
0.06		2	23							25
0.125	2	18	31	135			1			187
0.25		1	4	44	7					56
0.5				33	37	2				72
1	1				12	17				30
2				1		5	143	7		156
≥4							3	50	3	56
Total	137	42	67	213	56	24	147	58	3	747

Dark green highlight indicates matching MIC values; light green highlight indicates matching MIC values within +/- 1 dilution.

CLSI oral penicillin V resistance breakpoints used.

Table S21. Ceftriaxone predicted and phenotypic MIC accuracy on the Canadian validation dataset.

Predicted MIC (mg/L)	Phenotypic MIC (mg/L)							
	Susceptible			Intermediate	Resistant			
	0.125	0.25	0.5	1	2	4	8	Total
≤0.125	406	9	1					416
0.25		5						5
0.5	2							2
1			5	11				16
Total	408	14	6	11				439

Dark green highlight indicates matching MIC values; light green highlight indicates matching MIC values within +/- 1 dilution.

Table S21. Ceftriaxone predicted and phenotypic MIC accuracy on the USA-1 validation dataset.

Predicted MIC (mg/L)	Phenotypic MIC (mg/L)							
	Susceptible			Intermediate	Resistant			
	0.125	0.25	0.5	1	2	4	8	Total
≤0.125	477	17	1			1		496
0.25		1	6					7
0.5								
1	2	4	19	53	18	1		97
2								
≥4					1	4	2	7
Total	479	22	26	53	19	6	2	607

Dark green highlight indicates matching MIC values; light green highlight indicates matching MIC values within +/- 1 dilution.

Table S22. Ceftriaxone predicted and phenotypic MIC accuracy on the USA-2 validation dataset.

Predicted MIC (mg/L)	Phenotypic MIC (mg/L)							
	Susceptible			Intermediate	Resistant			
	0.125	0.25	0.5	1	2	4	8	Total
≤0.125	404	34	7	2	1			448
0.25	2	42	35	4				83
0.5	1	3	1					5
1	1	5	13	135	50			204
2								
≥4						4	3	7
Total	408	84	56	141	51	4	3	747

Dark green highlight indicates matching MIC values; light green highlight indicates matching MIC values within +/- 1 dilution.

Table S23. Erythromycin predicted and phenotypic MIC accuracy on the USA-1 validation dataset.

Predicted MIC (mg/L)	Phenotypic MIC (mg/L)									
	Susceptible		Intermediate	Resistant						
	≤0.125	0.25	0.5	1	2	4	8	16	≥32	Total
≤0.125	464			2				1	2	469
0.25	1									1
1	1									1
8	6	3	4	1	6	8	3	2		33
16	1					2	4	1		8
>=32	1					1			20	22
Total	474	3	4	3	6	11	7	4	22	534

Dark green highlight indicates matching MIC values; light green highlight indicates matching MIC values within +/- 1 dilution.

Table S24. Erythromycin predicted and phenotypic MIC accuracy on the USA-2 validation dataset.

Predicted MIC (mg/L)	Phenotypic MIC (mg/L)									
	Susceptible		Intermediate	Resistant						
	≤0.125	0.25	0.5	1	2	4	8	16	≥32	Total
0.125	321	5	1	1	1				1	330
1			1							1
8	1		1		8	54	84	30	2	180
16					3	3	11	11	6	34
>=32		1		3	2	3	4	4	185	202
Total	322	6	3	4	14	60	99	45	194	747

Dark green highlight indicates matching MIC values; light green highlight indicates matching MIC values within +/- 1 dilution.

Table S25. Clarithromycin predicted and phenotypic MIC accuracy on the Canadian validation dataset.

Predicted MIC (mg/L)	Phenotypic MIC (mg/L)												
	Susceptible				Intermediate	Resistant							
	≤0.03	0.06	0.12	0.25	0.5	1	2	4	8	16	32	≥64	Total
≤0.03	340	8	2	4									354
0.06													0
0.125													0
0.25													0
0.5													0
1													0
2						5	14	10	2	1	1		33
4					1		4	14	9	1	1		30
8													0
16													0
≥32								1	1	1		19	22
Total	341	8	2	4	1	5	18	25	12	3	2	19	439

Dark green highlight indicates matching MIC values; light green highlight indicates matching MIC values within +/- 1 dilution.

Table S26. Clindamycin predicted and phenotypic MIC accuracy on the Canadian validation dataset.

Predicted MIC (mg/L)	Phenotypic MIC (mg/L)								
	Susceptible		Intermediate	Resistant					
	≤0.125	0.25	0.5	1	2	32	64	128	Total
≤0.125	416								416
0.25									0
0.5	1								1
≥64				1	3	2	1	15	22
Total	417			1	3	2	1	15	439

Dark green highlight indicates matching MIC values; light green highlight indicates matching MIC values within +/- 1 dilution.

Table S27. Clindamycin predicted and phenotypic MIC accuracy on the USA-2 validation dataset.

Predicted MIC (mg/L)	Phenotypic MIC (mg/L)						
	Susceptible			Intermediate	Resistant		
	≤0.06	0.125	0.25	0.5	1	>2	Total
≤0.125	364	182		1		3	550
0.25		1	1				2
0.5						1	1
≥64	6*	6*	1*	3	1	177	194
Total	370	189	2	4	1	181	747

Dark green highlight indicates matching MIC values; light green highlight indicates matching MIC values within +/- 1 dilution.

* ermB gene present.

Table S28. Levofloxacin predicted and phenotypic MIC accuracy on the Canadian validation dataset.

Predicted MIC (mg/L)	Phenotypic MIC (mg/L)						
	Susceptible				Intermediate	Resistant	
	≤0.25	0.5	1	2	4	≥8	Total
≤1	1	37	384	11			433
2				4			4
4							
≥8						1	1
Error			1				1
Total	1	37	385	15		1	439

Dark green highlight indicates matching MIC values; light green highlight indicates matching MIC values within +/- 1 dilution.

Blue highlighted isolate has an assembly error with no gyrA gene present.

Total used in analysis n=438.

Table S29. Levofloxacin predicted and phenotypic MIC accuracy on the USA-2 validation dataset.

Predicted MIC (mg/L)	Phenotypic MIC (mg/L)							
	Susceptible				Intermediate	Resistant		
	≤0.25	0.5	1	2	4	8	≥16	Total
≤1		41	697	3				741
2			1	4				5
4								0
≥8						1		1
Total		41	698	7		1		747

Dark green highlight indicates matching MIC values; light green highlight indicates matching MIC values within +/- 1 dilution.

Table S30. Trimethoprim/sulfamethoxazole predicted and phenotypic MIC accuracy on the Canadian validation dataset.

Predicted MIC (mg/L)	Phenotypic MIC (mg/L)								
	Susceptible			Intermediate		Resistant			
	≤0.125	0.25	0.5	1	2	4	8	≥16	Total
0.25	179	188	9						376
1			11	15	8	1			35
2									0
≥4					1	12	12	3	28
Total	179	188	20	15	9	13	12	3	439

Dark green highlight indicates matching MIC values; light green highlight indicates matching MIC values within +/- 1 dilution.

Table S31. Trimethoprim/sulfamethoxazole predicted and phenotypic MIC accuracy on the USA-2 validation dataset.

Predicted MIC (mg/L)	Phenotypic MIC (mg/L)								
	Susceptible			Intermediate		Resistant			
	≤0.125	0.25	0.5	1	2	4	8	≥16	Total
0.25	102	306	25	2		2			437
0.5		3	12	8					23
1			10	53	68	9			140
≥4				1	6	140			147
Total	102	309	47	64	74	151			747

Dark green highlight indicates matching MIC values; light green highlight indicates matching MIC values within +/- 1 dilution.

Table S32. Chloramphenicol predicted and phenotypic MIC accuracy on the Canadian validation dataset.

Predicted MIC (mg/L)	Phenotypic MIC (mg/L)						
	Susceptible				Resistant		
	≤0.5	1	2	4	8	≥16	Total
≤4	1	43	270	110	5*	3*	432
≥8						7	7
Total	1	43	270	110	5	10	439

Dark green highlight indicates matching MIC values; light green highlight indicates matching MIC values within +/- 1 dilution.

Table S33. Chloramphenicol predicted and phenotypic MIC accuracy on the USA-1 validation dataset.

Predicted MIC (mg/L)	Phenotypic MIC (mg/L)						
	Susceptible				Resistant		
	≤0.5	1	2	4	8	≥16	Total
≤4		29	157	15			201
≥8							
Total		29	157	15			201

Dark green highlight indicates matching MIC values; light green highlight indicates matching MIC values within +/- 1 dilution.

Table S34. Chloramphenicol predicted and phenotypic MIC accuracy on the USA-2 validation dataset.

Predicted MIC (mg/L)	Phenotypic MIC (mg/L)						
	Susceptible				Resistant		
	≤0.5	1	2	4	8	≥16	Total
≤4			269	448	13		730
≥8					17		17
Total			269	448	30		747

Dark green highlight indicates matching MIC values; light green highlight indicates matching MIC values within +/- 1 dilution.

Table S35. Doxycycline predicted and phenotypic MIC accuracy on the Canadian validation dataset.

Predicted MIC (mg/L)	Phenotypic MIC (mg/L)							
	Susceptible	Intermediate	Resistant					
	≤0.25	0.5	1	2	4	8	≥16	Total
≤0.25	396		1*		1*			398
≥4	2**	2**	1	11	10	11	4	41
Total	398	2	2	12	11	11	4	439

Dark green highlight indicates matching MIC values; light green highlight indicates matching MIC values within +/- 1 dilution.

* tet genes not detected by ARG-ANNOT, CARD or ResFinder.

** tetM gene present.

Table S36. Tetracycline predicted and phenotypic MIC accuracy on the Canadian validation dataset.

Predicted MIC (mg/L)	Phenotypic MIC (mg/L)			
	Susceptible	Intermediate	Resistant	
	≤1	2	≥8	Total
≤1	12			12
≥16			1	1
Total	12	0	1	13

Dark green highlight indicates matching MIC values; light green highlight indicates matching MIC values within +/- 1 dilution.

Table S37. Tetracycline predicted and phenotypic MIC accuracy on the USA-1 validation dataset.

Predicted MIC (mg/L)	Phenotypic MIC (mg/L)			
	Susceptible	Intermediate	Resistant	
	≤1	2	≥4	Total
≤1	284	1	1*	286
≥16	4**		35	39
Total	288	1	36	325

Dark green highlight indicates matching MIC values; light green highlight indicates matching MIC values within +/- 1 dilution.

* tet genes not detected by ARG-ANNOT, CARD or ResFinder.

** tetM detected.

Table S38. Tetracycline predicted and phenotypic MIC accuracy on the USA-2 validation dataset.

Predicted MIC (mg/L)	Phenotypic MIC (mg/L)				
	Susceptible	Intermediate	Resistant		
	≤1	2	4	≥8	Total
≤1	508			8*	516
≥16	12**	1	3	215	231
Total	520	1	3	223	747

Dark green highlight indicates matching MIC values; light green highlight indicates matching MIC values within +/- 1 dilution.

* tet genes not detected by ARG-ANNOT, CARD or ResFinder.

** tetM detected.

Table S39. Minor interpretative errors of predicted and phenotypic MICs

Antimicrobial	Predicted/Phenotypic MIC Interpretation				Total
	I/S*	I/R	S/I	R/I	
Penicillin	32	1	56	31	120
Ceftriaxone	49	69	6	0	124
Erythromycin	0	0	1	6	7
Clindamycin	1	1	1	3	6
Clarithromycin	0	0	0	1	1
Levofloxacin	0	0	0	0	0
Trimeth/Sulfa	21	8	10	8	47
Chloramphenicol	0	0	0	0	0
Doxycylcine	0	0	0	2	2
Tetracycline	0	0	1	1	2
Total	103	80	75	51	309

* Interpretative classifications: S = Susceptible, I = Intermediate and R = Resistant.
